# Implementation of an SSI care bundle for patients undergoing emergency laparotomy

**DOI:** 10.1016/j.infpip.2026.100566

**Published:** 2026-07-10

**Authors:** Cara Swain, Oliver Brewster, Marcus Quinn, James Thornton, Dawn Gane, Jodie Rogers, Anne Pullyblank

**Affiliations:** a Academic Department of Military Surgery and Trauma, Royal Centre for Defence Medicine, Birmingham, UK; b Department of Learning, Informatics, Management and Ethics, Karolinska Institutet, Stockholm, Sweden; c Great Western Hospitals NHS Trust, Swindon, UK; d North Bristol NHS Trust, Southmead, Bristol, UK; e Royal Cornwall Hospitals NHS Trust, Truro, UK

**Keywords:** Surgical site infection, Emergency laparotomy, Quality improvement

## Abstract

**Background:**

Surgical site infection (SSI) is common after emergency laparotomy (EL) due to contamination of the surgical field, yet most hospitals are unaware of their SSI rate. Most studies have focused on elective surgery where the surgical environment can be standardized easily.

**Methods:**

This prospective quality improvement study, with continuous SSI surveillance over 4 years, aimed to establish a reliable method for measuring post-EL SSI and to evaluate the impact of a care bundle (2% chlorhexidine skin preparation, second antibiotic dose after 4 h, use of a wound protector, antibacterial sutures, glove change and povidone iodine wound wash) to reduce SSI.

**Results:**

There were 176 patients pre intervention and 501 patients post intervention. The baseline SSI rate was 32.1%: 23.1% for clean wounds, 24.5% for clean-contaminated wounds, and 45.0% for dirty wounds. Post intervention, the overall SSI rate decreased to 18.1% (*P*=0.002): 11.8% for clean wounds (*P*=0.275), 15.1% for clean-contaminated wounds (*P*=0.121), and 25.6% for dirty wounds (*P*=0.021).

**Discussion:**

Patients undergoing EL are complex. Measuring SSI after EL is challenging due to high mortality, use of laparostomy, and longer length of stay, meaning in-hospital surveillance for patients staying >30 days is also required. Implementing behavioural change in a diverse group of surgeons in the emergency setting, during a pandemic in a National Health service hospital, was difficult.

**Conclusion:**

This study demonstrated a 44% reduction in post-EL SSI following introduction of a care bundle, highlighting the value of structured quality improvement in emergency surgery. SSI measurement following EL is challenging but feasible.

## Background

Surgical site infection (SSI) affects 12.3% of patients worldwide [1] and accounted for 15.7% of all healthcare-associated infections in England in 2011 [2]; an estimated 100,965 cases annually [3]. SSI is a common complication after emergency laparotomy (EL), but EL is not included in current National Health Service (NHS) national surveillance [4]. SSI increases the risk of wound dehiscence [5], incisional hernia [6], prolonged inpatient admission [7,8], re-admission [9], further surgical procedures and intensive care support [10], and can contribute to patient death [11] with an associated high cost [12]. Bowel surgery costs hospitals £119 million per annum [3], with the excess cost per infection ranging from £3746 to £10,523 [3,7,13], and £3535 after open surgery [14]. Despite this, most hospitals do not know their SSI rate. The Getting It Right First Time (GIRFT) 2017 report stated that only four of 50 participating hospitals were able to give a reliable report of their wound infection rate for elective general surgery [15].

Hospitals are required to report SSI following some elective procedures, but this does not include general surgery or emergency procedures [4]. The standard measure for SSI in England is patient-reported at 30 days after surgery using the UK Health Security Agency (UKHSA) wound healing questionnaire. Postdischarge surveillance is important, as reporting which relies on inpatient SSI or re-admissions alone will underestimate the SSI rate [9]; 85% of cases of SSI after open surgery present in the community [14]. SSI commonly presents 11 days following elective colorectal surgery [16,17] and 18 days following open surgery [14]. The median length of stay (LOS) following EL is 11 days (interquartile range (IQR) 6–19 days), increasing to 31 days (IQR 18–55 days) if there is an unplanned return to theatre, which occurs in 5.5% of cases [18].

EL is a procedure performed for a heterogenous group of patients for a diverse range of pathologies, such as intestinal obstruction, perforation and peritonitis. Patients are often frail with limited physiological reserve. The in-hospital mortality rate is 9.3% [18], increasing to 15.3% in patients aged >65 years [19], compared with 1–2% for elective procedures. EL carries a higher risk of postoperative complications [20], and SSI is more likely after dirty procedures [1] where the surgical site is already contaminated.

Use of a care bundle has been shown to reduce the SSI rate by 40–50% [17,21,22] in patients undergoing elective colorectal surgery and 7.6% in patients undergoing EL [23]. There is no consensus on what constitutes a care bundle for SSI, except inclusion of three or more interventions. The analysis of efficacy is limited by insufficient data [24], and the effect is inconsistent in randomized controlled trials, with larger intervention bundles not associated with a more significant effect [22,25,26]. Some perioperative factors, such as maintaining normothermia and controlling blood glucose, are addressed in the World Health Organization (WHO) perioperative checklist [21,23,27,28]. Implementation of any care bundle requires behavioural and systemic change at individual and team level.

### Local problem

Prior to this study, SSI surveillance was limited to elective procedures. A four-point evidence-based care bundle introduced to elective colorectal surgery in 2013 reduced the SSI rate by 50% [17]. Results were replicated across the West of England region, reducing the SSI rate from 18% to 9.5% across seven hospitals [29]. All bundle elements are in the WHO [30] and National Institute for Clinical Excellence (NICE) [31] guidance for reducing SSI.

Measurement of SSI following EL is challenging due to higher rates of early mortality (≤30 days after surgery), a longer LOS, and a proportion of patients who are managed with laparostomy, some of whom will have wounds that heal by secondary intention. As a significant proportion of patients remain in hospital 30 days after surgery, patient-reported SSI using the UKHSA questionnaire will not capture the total incidence of SSI without additional in-hospital surveillance.

### Intended improvement

This study aimed to adapt the elective colorectal surgery care bundle for EL with the addition of two elements: povidone iodine wound wash (included in the WHO guidance, although with weak supporting evidence); and a change of surgical gloves prior to skin closure. Glove change appeared rational as the surgical field is frequently contaminated in EL, and this was subsequently supported by the Cheetah trial [32]. The adapted SSI care bundle for EL included:

• 2% chlorhexidine isopropyl alcohol skin preparation;
• second (repeat) dose of antibiotics after 4 hours of operative time;
• use of a dual ring wound protector (WP);
• antibacterial sutures for deep and superficial closure;
• theatre team to change gloves prior to skin closure; and
• povidone iodine wash to surgical wound.

The goal was to reduce the SSI rate through consistent application of evidence-based practices. Prior to implementing the EL care bundle, the authors confirmed high compliance with the SSI care bundle in the WHO checklist (i.e. appropriate hair removal, on-time antibiotics, maintenance of normothermia and glucose control in diabetic patients). The study questions were: Can SSI be measured reliably after EL? Can an adapted care bundle reduce the SSI rate effectively in this high-risk patient population?

### Setting

This quality improvement project was conducted at an 800-bed teaching hospital and major trauma centre in south-west England serving a population of approximately 750,000. EL for gastrointestinal conditions is performed by a team of 14 upper gastrointestinal and colorectal surgeons, with approximately 200 procedures annually. EL is delivered 24/7, typically in a designated emergency theatre, although other theatres may be used during periods of clinical pressure. The variability in operating environments and multi-disciplinary teams, including rotating nursing staff, posed challenges to standardizing surgical practice and implementing interventions consistently.

## Planning the intervention

The adapted care bundle was co-designed with clinical teams following review of the evidence base for additional bundle elements. Interventions had to be affordable; this excluded the routine use of negative pressure dressings.

Implementation faced logistical challenges; for example, unlike elective surgery, there was no nursing team to support SSI surveillance. Patients were identified from the enhanced recovery database of EL and cross-referenced with the National Emergency Laparotomy Audit (NELA) database. Dissemination of patient questionnaires was implemented monthly by the lead consultant, supported by resident doctors and a medical secretary. Strategies were developed to capture SSI data for patients with LOS >30 days. SSI was measured on a 4-weekly basis, 1 month in arrears, as a three-stage process:

• UKHSA questionnaire by post. The 30-day patient questionnaire was sent to patients who had been discharged 30 days after surgery (i.e. all patients who underwent EL in March were sent a questionnaire at the end of April). The UKHSA questionnaire uses questions which classifies superficial SSI dependent on groups of symptoms being present (Table I). If SSI was reported by the patient, it was classified as Criteria 1, 2 or 3. In a minority of cases, patients discharged within 30 days of surgery were known to have developed SSI so were not sent a questionnaire.

**Table I:** Criteria for characterizing surgical site infection

Criteria	Clinical signs
1	Discharge or leakage of pus from any part of the wound AND antibiotics prescribed
2	At least two clinical signs: • Pain or soreness in addition to discomfort following the operation • Redness or inflammation spreading from the edges of the wound • Area around the wound felt to be warmer/hotter than the surrounding skin • Area around the wound became swollen AND edges of any part of the wound separated or gaped open
3	At least two clinical signs: • Pain or soreness in addition to discomfort following the operation • Redness or inflammation spreading from the edges of the wound • Area around the wound felt to be warmer/hotter than the surrounding skin • Area around the wound became swollen AND antibiotics prescribed
• Clinical assessment. If EL patients were still inpatients at the time of data collection, SSI was assessed by contemporaneous clinical assessment.
• Documentation review. For patients who had been discharged prior to the time of monthly review, but remained in hospital >30 days after surgery, SSI was determined by retrospective review of both the patient's discharge summary and electronic patient record for documented clinical diagnosis of SSI.

The SSI care bundle was launched in February 2020 through departmental meetings. Theatre staff were educated on bundle components and indications. There had been longstanding quality improvement work on SSI after elective surgery at the study hospital, meaning many staff members understood the methodology. Elective theatre nursing teams familiar with the colorectal bundle supported emergency theatre teams, and a theatre champion supported bundle compliance, reinforced by posters. Compliance was initially recorded on paper by theatre nursing staff, and later integrated into the electronic theatre system. The SSI rate and data were reported to the teams regularly.

### Ethical considerations

This quality improvement project was registered locally and was exempt from formal ethics review.

### Data collection

Data were collected continuously from March 2019 to February 2023 for all patients undergoing EL. Demographic and clinical data were recorded retrospectively in a bespoke spreadsheet, including: patient demographics (identifier, age, gender); operation date; procedure performed; degree of contamination (localized/generalized); laparostomy at index procedure; inclusion of stoma formation; and date of discharge or death.

The degree of contamination was identified from NELA. For cases which had not been included on the NELA database, the operation notes were reviewed for statements made regarding contamination. The final Office of Population Censuses and Surveys (OPCS) categorization was based on operative findings and the procedure performed (e.g. a patient with a ‘clean’ abdomen at initial laparotomy would be coded ‘clean-contaminated’ if they underwent bowel resection).

To standardize the cohort population to primary laparotomy for gastrointestinal conditions, patients undergoing EL for trauma or non-gastrointestinal problems (e.g. vascular, gynaecology or urology), complications of elective surgery, trephine stoma surgery, laparoscopic procedures, and hernia repairs without bowel resection were excluded. The small number of EL patients who had subsequent re-operations were not excluded. Patients with open wounds left to granulate or those with an enterocutaneous fistula were excluded; if a closed wound dehisced, this would be a symptom suggesting an SSI diagnosis. Patients who died within 30 days of surgery were also excluded; patients who died >30 days after surgery had their SSI status recorded.

### Data analysis

The monthly incidence of SSI can vary widely depending on numbers of eligible EL and questionnaire response rates. Data analysis was performed using Excel (Microsoft Corp., Redmond, WA, USA); a run chart was used to visualize the monthly SSI rate using NHS software [33]. A run chart is a quality improvement tool to monitor processes over time (x-axis), where the central line (y-axis) denotes the mean average. Common cause variation is the expected month-to-month noise present in any process. A shift of seven to nine or more consecutive data points on one side of the centre line indicates that a change not related to natural variation has occurred. With monthly data, six data points can be acceptable to demonstrate a shift. This is called ‘special cause variation’ and, if the timing aligns, can be attributed to the intervention.

A secondary outcome was SSI by contamination status between pre- and postintervention EL, analysed using Chi-squared test with significance set at *P*=0.05.

## Results

**Figure 1:**
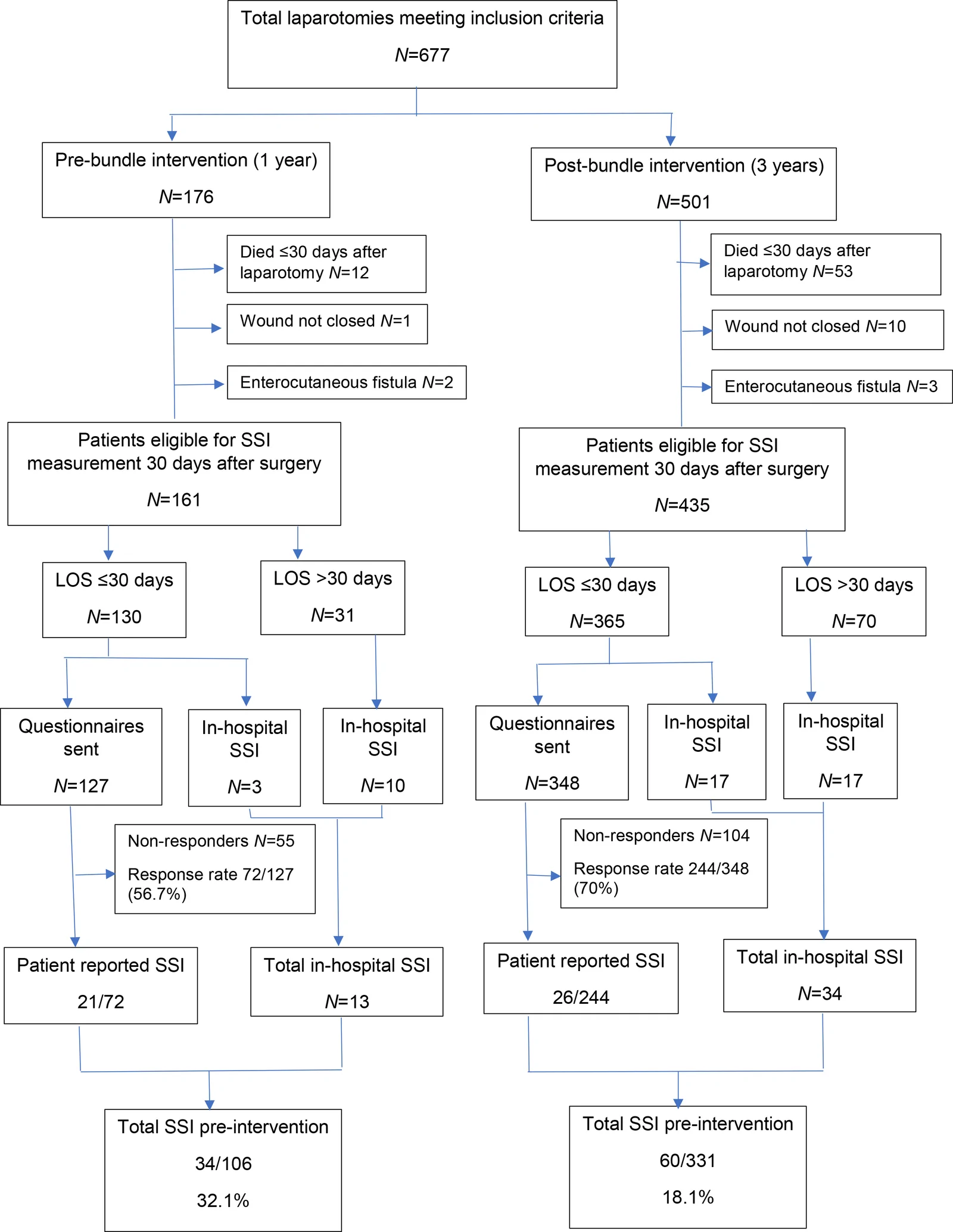
Consort diagram. LOS, length of stay; SSI, surgical site infection.

**Figure 2:**
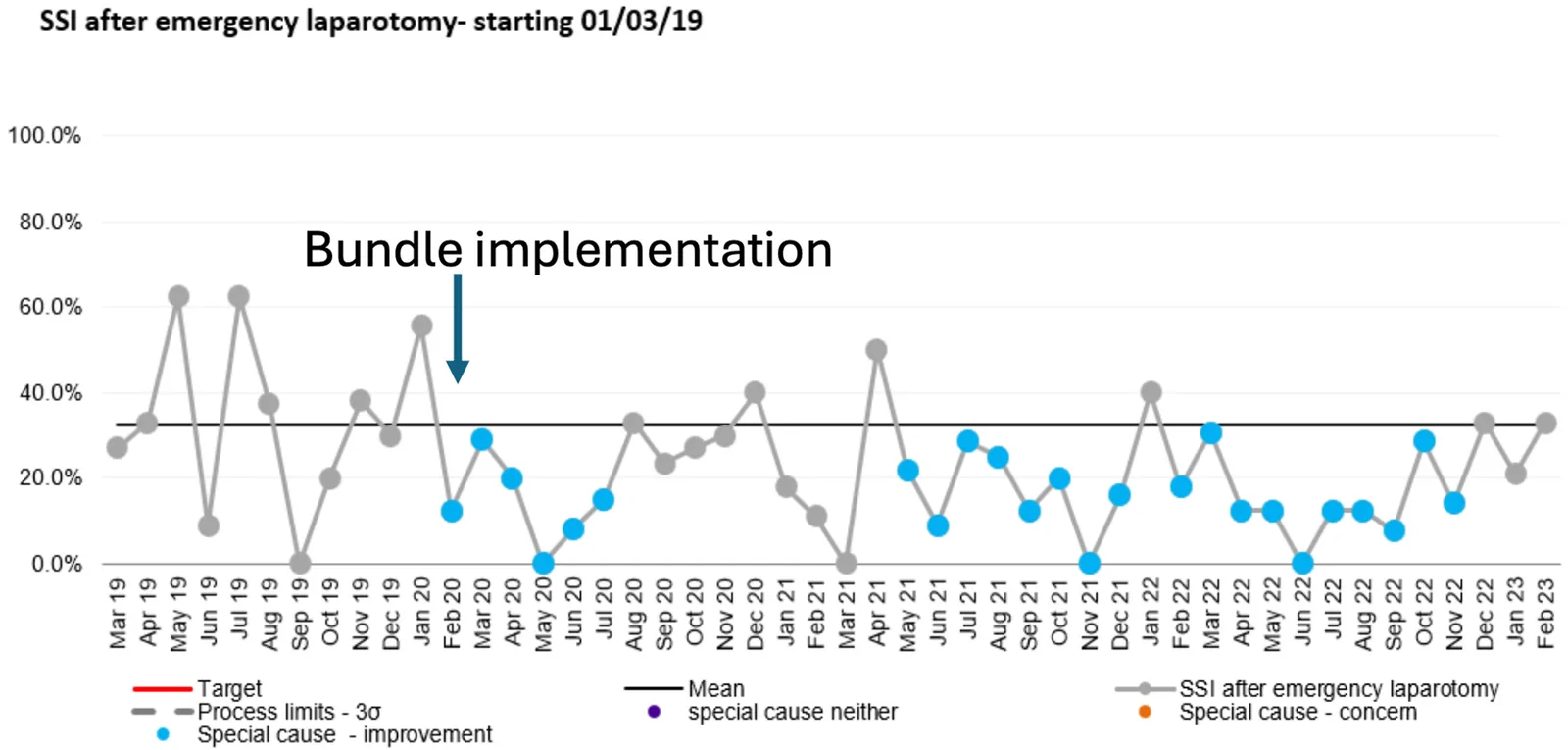
Run chart. Time is reported on the x-axis. The central line denotes the mean average. A run is defined as one or more consecutive data points on the same side of the average. Blue data points below the mean denote a significant shift if this occurs in six consecutive data points or more. SSI, surgical site infection.

In total, 677 patients underwent EL during the study period. After exclusions, the final analysed cohort was 161 pre-intervention patients and 435 postintervention patients. The pre- and post-population demographics are described in Table II with pre- and postintervention SSI rates demonstrated in the Consort diagram (Figure 1). Continuous data for SSI rate are shown in the run chart (Figure 2), and demonstrate special cause improvement despite the inherent variability of the SSI rate. Where the SSI rate was particularly high or zero, this tended to be associated with inclusion of a low number of patients for that month.

**Table II:** Overall study population demographics

	Pre intervention	Post intervention
Total number of patients	176	501
Male:female	1:1.26 (78:98)	1:1.06 (240:256)
Age, years (mean)	63.0 (range 17–94)	63.6 (17–93)
Average LOS, days (mean)	22.6 (range 1–215)	18.6 (range 1–161)
Total number of deaths	9.1% (*N*=16)	12.0% (*N*=60)
Death ≤30 days after surgery	6.8% (*N*=12)	10.6% (*N*=53)
Death >30 days after surgery	2.3% (*N*=4)	1.4% (*N*=7)
Laparostomy	11.9% (21/176)	12.0% (60/501)
Enterocutaneous fistula	2	3
Procedure		
Adhesiolysis	17.0% (*N*=30)	16.2% (*N*=81)
Gastric surgery	2.3% (*N*=4)	4.8% (*N*=24)
Left-sided colonic resection	26.1% (*N*=46)	25.1% (*N*=126)
Right-sided colonic resection	19.9% (*N*=35)	17.6% (*N*=88)
Small bowel resection	27.3% (*N*=48)	26.9% (*N*=135)
No resection	4% (*N*=7)	6.6% (*N*=33)
Stoma formation	2.8% (*N*=5)	2.8% (*N*=14)
Contamination status		
Clean	16.5% (*N*=29)	18.6% (*N*=93)
Clean-contaminated	47.7% (*N*=84)	43.3% (*N*=217)
Dirty	35.8% (*N*=63)	38.1% (*N*=191)

LOS, length of stay.

Pre-intervention, 127/161 patients were sent a questionnaire and 72 responded (56.7%). Three patients were known to have an SSI prior to discharge and were not sent a questionnaire; 31 patients had LOS >30 days, of whom four patients died >30 days after surgery. SSI was reported by 21 patients who responded to the questionnaire, and diagnosed in 13 patients by in-hospital surveillance. Exclusion of non-responders resulted in a data completion rate of 65.8%.

Post intervention, 348/435 patients were sent a questionnaire, with a response rate of 70.1%. Seventeen patients were known to have an SSI prior to discharge and were not sent a questionnaire. Seventy patients had in-hospital surveillance due to LOS >30 days, including seven who died >30 days after surgery. This resulted in overall data completion of 76.1%. SSI was reported by 26 patients who responded to the questionnaire, and was diagnosed in 34 patients by in-hospital surveillance. After introduction of the care bundle, the overall SSI rate reduced from 32.1% to 18.1% (*P*=0.002).

The overall mortality rate was 11.2% (76/677), and most patients died within 30 days of surgery (median 7 days, range 0–26 days); only 1.6% (*N*=11) of patients died >30 days after surgery. The laparostomy rate was 12.0% (81/677), with an associated SSI rate of 24%.

Patients with LOS >30 days (16.9%) had a higher baseline SSI rate of 32.3%, and the care bundle appeared to be less effective, with a postintervention SSI rate of 28.6%. These patients had more severe wound problems (e.g. dehiscence). If LOS was ≤30 days, the baseline SSI rate was 32%, reducing to 15.3% post intervention. Fewer than 1% of patients were re-admitted due to an SSI.

When classified according to degree of contamination as per OPCS coding, 19.3% of procedures were clean, 47.5% were clean-contaminated, and 33.3% were dirty. The baseline SSI rates were 23.1% (3/13) for clean wounds, 24.5% (13/53) for clean-contaminated wounds, and 45.0% (18/40) for dirty wounds. Postintervention SSI rates reduced, although not significantly, to 11.8% for clean wounds (*P*=0.275) and 15.1% for clean-contaminated wounds (*P*=0.121), and decreased significantly to 25.6% for dirty wounds (*P*=0.021).

### SSI care bundle compliance

There was high compliance with the care bundle across most elements (Table III):

• 100% for chlorhexidine skin preparation;
• 90% for use of antibacterial deep fascial sutures;
• 89.5% for antibacterial superficial skin sutures;
• 87.2% for povidone iodine wound wash; and
• 77.6% for glove change prior to closure.

**Table III:** Compliance with surgical site infection care bundle elements (by year)

	Year 1	Year 2	Year 3	Overall %
2% chlorhexidine	100%	100%	100%	100%
Wound protector	48.9% (43/88)	45.6% (36/79)	46.9% (60/128)	47.1%
Second dose of antibiotics	25.0% (2/8)	50.0% (3/6)	100.0% (5/5)	52.6%
Glove change	75.3% (64/85)	79.5% (58/73)	79.4% (27/34)	77.6%
Povidone iodine wash	84.5% (71/84)	93.1% (67/72)	81.3% (26/32)	87.2%
Antibacterial sutures				
Fascia	89.4% (76/85)	87.2% (68/78)	92.1% (117/127)	90.0%
Skin	86.6% (71/82)	91.1% (72/79)	90.4% (113/125)	89.5%

Compliance was measured for all patients and included patients who were subsequently excluded. Bundle compliance was available for 68% of the final dataset where SSI data were available.

Overall compliance with antibiotic administration was near 100%. Only 19 patients underwent EL exceeding 4 h. Although only 10 patients were documented as having a second dose of antibiotics, on review, many patients were either treated for sepsis pre-operatively and/or prescribed antibiotics with a long half-life (e.g. piperacillin/tazobactam or meropenem), limiting the reliability of this measure.

Compliance with use of a dual ring WP was lower at 47.5% (50% in the final dataset), and was the primary reason why the entire care bundle was not delivered. Most patients had the remainder of the care bundle. Had a WP been used consistently, only an additional eight cases were non-compliant with sutures (*N*=6) and/or glove change (*N*=2). Forty-seven percent of patients had the whole care bundle delivered; only two patients received no element of the care bundle at all. The SSI rate was 15% with WP use and 24.3% without WP use.

## Discussion

This quality improvement initiative demonstrated that implementing a targeted care bundle for EL can lead to a reduction in the SSI rate from 32.1% to 18.1%, particularly in dirty procedures. Despite challenges in standardizing care across diverse surgical teams and patient presentations, high compliance with most bundle elements was achieved. A reduction in the SSI rate was sustained over 3 years, suggesting that the impact of the care bundle is immediate and durable when compliance is high. The study also highlighted the importance of combining patient-reported outcomes with in-hospital surveillance to capture the incidence of SSI accurately.

Care bundles are known to be effective in reducing the SSI rate after elective surgery [17,21,34,35]; however, there is little evidence for their use after EL [24]. In a meta-analysis of 11 studies deemed eligible for inclusion [27], just six measured the 30-day SSI rate, and of the seven included studies, only two compared the SSI rate pre and post intervention. Of these, Phelan *et al.* [23] included chlorhexidine isopropyl alcohol skin preparation, antibacterial sutures and a surgical team glove change, and Yamamoto *et al.* [36] included antibacterial sutures alone from the care bundle implemented in the present study.

A UK study by Phelan *et al.* reported a reduction in the SSI rate from 29.3% to 21.7%, but this was not significant [23]. This is probably due to low numbers (99 patients pre intervention and 163 post intervention), short duration (only 6 months post implementation), and inclusion of both emergency and elective patients. Their care bundle consisted of three pre-operative, five intra-operative and three postoperative elements, and compliance with bundle elements was not measured.

Since completion of the present study, a study has been published describing the use of a similar care bundle over the same time period (2020–2023) [37]. Isand *et al.* [37] implemented an 11-point care bundle which included the four elements of the WHO checklist as well as the six elements of the present care bundle. Of note, they allowed a 3-month ‘settling-in period’ where the care bundle was used but data were not collected; the present data included this settling-in period, which also corresponded with the onset of the coronavirus disease 2019 (COVID-19) pandemic. The study by Isand *et al.* [37] was restricted to a small group of dedicated emergency surgeons. They excluded patients who did not receive the care bundle from the analysis, and adherence to individual bundle elements was not assessed formally. Despite this, their results are very similar to the present results, with a pre-bundle SSI rate of 32.2% and a post-bundle SSI rate of 20.5% [37]. The present authors' real-world evidence supports their findings, suggesting that the effect of the care bundle is real, despite the limitations of this quality improvement study.

Managing patients with physiological derangement and/or excessive contamination with an open abdomen has become common in EL. The study centre has a laparostomy rate of 9.5%, with 85.5% achieving primary closure 2.1 days after the initial procedure [38]. Given that 51% of patients managed with laparostomy had dirty wounds and only 9% had clean wounds, the observed SSI rate of 24% in these patients may be lower than expected.

When analysed by contamination status, the SSI rate increased with degree of contamination, and the care bundle was found to be most effective for dirty wounds, reducing the SSI rate from 45.0% to 25.6%. Phelan *et al.* [23] also found a significant reduction in the SSI rate in patients with dirty wounds. The reduction in SSI rate in clean wounds (from 23.1% to 11.8%) and clean-contaminated wounds (from 24.5% to 15.1%) was not significant, likely due to small numbers. Compliance with glove changing was lower at 60% (vs 76% overall) when the operation was clean, suggesting that surgeons may not have considered the need to change gloves or use a WP if there was no visible contamination. In contrast, compliance for dirty operations was 45% for WP use and 82.5% for glove changes. If the care bundle really is less effective for clean wounds, this may be because bundle elements which target enteral bacteria are less relevant in the absence of contamination.

### Challenges and limitations

Whilst all elements in this four-part care bundle for elective colorectal surgery were proven interventions [16,17,29] aligning with NICE and WHO guidance, not all were applicable to EL as few EL procedures exceed 4 h, making the requirement for a second dose of antibiotics largely redundant. In addition, recording of a second dose of antibiotic was unreliable as the questionnaire did not ask whether it was required, only whether it was given. 2% chlorhexidine isopropyl alcohol skin preparation was already used regularly with high compliance, whereas this was a shift in practice for elective surgery.

Compliance with dual ring WP use was low (50%), especially in clean surgery (25%). Lower compliance with WP use was observed in both previous studies in elective colorectal surgery [17,29]. A lack of availability of extra-large WPs likely limited use, especially in laparotomies for obese patients, along with the lack of effectiveness described in the ROSSINI trial [39] influencing surgical practice. The SSI rate was higher if a WP was not used, suggesting that WP use had an effect as this was a group with fewer contaminated surgeries. WPs can be difficult to use, especially in the presence of adhesions where it may not be possible to insert the WP under the wound edge; for clean cases, adhesiolysis was the typical index procedure. A meta-analysis of 22 eligible randomized controlled trials [40] indicated evidence to support the use of WPs to reduce superficial SSI in clean-contaminated and contaminated abdominal surgeries, but not in clean cases.

It was also difficult to change the practice of a small minority of surgeons from use of metal clips to antibacterial sutures for wound closure. Empowering theatre staff to challenge this and offer antibacterial sutures as an alternative was effective at almost eliminating this practice.

A limitation of this analysis relates to the volume and accuracy of compliance data. Overall, compliance data were obtained for almost 70% of patients included in the final analysis. Compliance was recorded for many excluded patients, suggesting that the practice of using the care bundle was adopted widely.

Following up emergency patients is more difficult than following up patients who have undergone elective surgery, among whom this can be carried out almost exclusively by postdischarge surveillance. SSI could only be measured in patients who were alive and had fascial closure by 30 days after surgery. For EL, 16.9% of patients were still in hospital 30 days after surgery, 9.6% died before 30 postoperative days, and 1.6% did not have a closed wound; this required a hybrid approach with in-hospital surveillance for patients remaining in hospital 30 days after surgery. The variable questionnaire response rate from 56% pre intervention to 70% post intervention is also a limitation. In previous projects, the authors have telephoned non-responders, but this requires personnel resources that were not available in this study.

Finally, the care bundle was implemented in February 2020 just prior to the UK COVID-19 lockdown. Inconsistent theatre environments and staff unfamiliarity due to pandemic-related disruption may explain why the reduction was not sustained initially.

### Lessons learned

• Bundle design – the care bundle is simple and evidence-based. In retrospect, it could have been further tailored to an emergency setting by excluding a second dose of antibiotics afer 4 h.
• Staff engagement – empowering theatre staff to prompt use of the care bundle and record compliance is key to success.
• Measurement strategy – a hybrid approach is required, combining patient-reported outcomes and in-hospital surveillance.

In conclusion, this study demonstrated a reliable dual approach to measure SSI after EL. Despite the limitations of running a quality improvement project in an emergency NHS setting, this study demonstrated that reliable implementation of a low-cost, evidence-based care bundle can lead to a 44% reduction in the SSI rate, especially in contaminated cases. However, the SSI rate remains unacceptably high, and further work is required to improve outcomes for patients.

## CRediT authorship contribution statement

**C. Swain:** Writing – review & editing, Writing – original draft, Methodology, Formal analysis, Data curation. **O. Brewster:** Writing – review & editing, Formal analysis, Data curation. **M. Quinn:** Writing – review & editing, Formal analysis, Data curation. **J. Thornton:** Writing – review & editing, Data curation. **D. Gane:** Writing – review & editing, Project administration, Data curation. **J. Rogers:** Writing – review & editing, Project administration, Data curation. **A. Pullyblank:** Writing – review & editing, Writing – original draft, Supervision, Project administration, Methodology, Investigation, Formal analysis, Data curation, Conceptualization.

## Ethics statement

This quality improvement project was registered and approved locally by the North Bristol NHS Trust Quality and Audit Committee, and considered to be exempt from formal ethics review.

## Funding sources

None.

## Conflicts of interest statement

None declared.
